# Flaxseed Prevents Leukocyte and Platelet Adhesion to Endothelial Cells in Experimental Atherosclerosis by Reducing sVCAM-1 and vWF

**DOI:** 10.1155/2013/303950

**Published:** 2013-12-30

**Authors:** Raluca Ecaterina Haliga, Roxana Irina Iancu, Doina Butcovan, Veronica Mocanu

**Affiliations:** ^1^Departments of Pathophysiology, General Pathology and Morphopathology, Grigore T. Popa University of Medicine and Pharmacy, 16 Universitatii Street, 700115 Iasi, Romania; ^2^Department of General Pathology, Grigore T. Popa University of Medicine and Pharmacy, 16 Universitatii Street, 700115 Iasi, Romania

## Abstract

We studied the possible effect of flaxseed to prevent leukocytes and platelets adhesion to endothelial cells and to reduce soluble adhesion molecules (sVCAM-1) and endothelial integrity markers (vWF) in ovariectomized rats fed a high-fat diet. Forty-two female Wistar rats were either sham-operated or ovariectomized and randomly assigned for 36 weeks to three different diets: (1) low-fat diet (8% energy as fat); (2) high-fat diet (40% energy as fat, lard based, lard group); (3) high-fat diet enriched with ground flaxseed 15 g/100 g of food (40% energy as fat, lard + flaxseed group). The ovariectomized rats fed with lard + flaxseeds had significantly lower serum concentrations of sVCAM and vWF, reduced platelet adhesiveness, and lower extent of platelet and leukocyte adherence to endothelium in the histological evaluation of the aorta as compared to Ovx + lard group. In our study, high dose of ground flaxseed incorporated to lard-based diet prevented the progression of atherosclerotic lesions in estrogen deficiency rats by decreasing platelet and endothelium reactivity. Assessment of platelet adhesion, serum soluble adhesion molecule sVCAM, and endothelium integrity molecule vWF could be useful to detect the risk for atherosclerotic lesions in estrogen deficiency states and to estimate the effect of flaxseed supplementation.

## 1. Introduction

In recent years, the use of foods with health protective effects, named functional foods, has received considerable attention for reducing cardiovascular disease (CVD) risk, one of these being flaxseed [1]. Flaxseeds are known to contain 35–40% fat, of which 55% is represented by *α*-linolenic acid (n3 polyunsaturated fatty acids, n3 PUFA) and 15–18% linoleic acid (n6 polyunsaturated fatty acids PUFA) and their metabolites. In addition to being the richest plant source of *α*-linolenic acid (ALA; 50–62% of flaxseed oil or *≈*22% of whole flaxseed) and lignans (mainly secoisolariciresinol diglucoside, SDG), which have phytoestrogen properties, flaxseed is an essential source of dietary fiber (28% by weight), of which 25% is in the soluble form [2]. All of these components could positively influence women's CVD risk profile [3, 4]. In our current diet, the ratio n6 PUFA/n3 PUFA is 20–30/1, comparing to a ratio of 1–4/1 in the period when the human genetic code was established in relation to the type of diet.

Previous studies [1, 3] have demonstrated that flaxseed is beneficial in reducing hypercholesterolemia and progression of atherosclerotic lesions in ovarian hormone deficiency. The available data sustain that the cardioprotective properties of flaxseed are due not only to hypocholesterolemic effects but also to other potential mechanisms, such as antioxidative, anti-inflammatory, and antithrombotic effects. Few studies have been conducted in order to assess the effects of flaxseed components on platelet and endothelial dysfunction [5].

The adhesion of both leukocytes and platelets to endothelial cells has been implicated in the progression of atherosclerosis and thrombus formation [6, 7]. Endothelial cells express adhesion molecules such as P-selectin, E-selectin, intercellular adhesion molecule 1 (ICAM-1), and vascular cell adhesion molecule 1 (VCAM-1) on the cell surface that are involved in leukocyte recruitment and platelet adhesion during thrombosis and inflammation [6]. In addition, endothelial cells synthesize plasma proteins such as von Willebrand factor (vWF) for platelet adhesion in thrombosis and soluble molecules such as E-selectin and thrombomodulin (TM) [8]. When the vascular endothelium encounters inflammatory stimuli, it undergoes several changes, including the upregulation of surface and soluble cell adhesion molecules and the release of cytokines. This process, termed endothelial activation, can be triggered by a variety of inflammatory stimuli encountered in the blood, including oxidized LDL, free radical species, lipopolysaccharide (LPS), and cytokines, such as tumor necrosis factor *α* (TNF *α*).

The activated endothelium plays an integral role in the development of atherosclerosis. Circulating monocytes are attracted to the endothelium by chemokines, bind to the adhesion molecules, adhere, and transmigrate to the subendothelial space, where they become macrophages, scavenge oxidized LDL, become foam cells, and contribute to the development of the fatty streak in the early stage of atherosclerosis [9].

The purpose of this study was to determine the possible effect of flaxseed to prevent leukocytes and platelets adhesion to endothelial cells and to reduce soluble adhesion molecules (sVCAM-1) and endothelial integrity markers (vWF) in ovariectomized rats fed a high-fat diet.

## 2. Materials and Methods

### 2.1. Animals

Forty-two female Wistar rats (14 weeks old, weight 200 ± 20 g) were used in the experiment. The rats were purchased from the animal farm of the discipline of Pathophysiology, Grigore T. Popa University of Medicine and Pharmacy, Iasi, Romania.

All experimental procedures used in this study were in strict accordance with international ethical regulations and were approved by the Medical Ethics Committee of the Grigore T. Popa University of Medicine and Pharmacy. The experiment respected as well the instructions of the Guidelines on the Care and Use of Animals for Scientific Purposes, National Advisory committal for Laboratory Animal Research, 2004.

The animals were anaesthetized with an intraperitoneal injection of a mixture of Ketamine, doses of 100 mg/kg bodyweight and Xylazine, doses of 10 mg/kg bodyweight. Half of the rats (*n* = 21) were subjected to bilateral ovariectomy (Ovx using the dorsolateral approach). The remaining animals (*n* = 21) were subjected to sham surgery (Sham), during which the ovaries were exteriorized but replaced intact.

### 2.2. Diets

The rats were kept in standard laboratory conditions, with a controlled temperature (20 ± 2°C) and a 12 h light/12 h dark cycle. The rats were provided with laboratory chow 20 g food/rat/day and tap water ad libitum. Each of the two groups (Ovx and Sham) were randomly assigned for 36 weeks to three different diets: (1) low-fat diet (8% energy as fat, deficient in ALA, control); (2) high-fat diet (40% energy as fat, lard based, lard group); (3) high-fat diet enriched with ground flaxseed 15 g/100 g of food, rich in alpha-linolenic acid, ALA (lard + flaxseed group).

Diets had similar carbohydrate, total fiber, protein, and fat content (Table 1).

### 2.3. Animal Necropsy and Processing of Samples

After 36 weeks, the animals were sacrificed, by thiopental anesthesia (1 mL/100 g body weight from 0.01% solution), followed by opening the chest and collecting the blood by cardiac puncture. Blood samples were collected using sodium citrate as anticoagulant buffer, blood/citrate ratio of 9 : 1, or without anticoagulant. The anticoagulated blood was centrifuged (200 ×g) for 10 min and the platelet-rich plasma (PRP) was removed and kept at room temperature for use within 4 h. Aliquots of serum were frozen and kept at −80°C for later analysis.

### 2.4. Parameters of Endothelial Dysfunction

Serum VCAM-1 was measured by ELISA method for quantitative evaluation of human sVCAM-1 (Bender Medical System) [10].

Serum vWF was measured by an immunoenzymatic “sandwich" method for vWF antigen (Life Therapeutics) [11].

### 2.5. Platelet Functions: Aggregation and Adhesion

Platelet aggregation to ADP 10 *μ*M final concentration was performed using a kinetic microplate reader (Tecan Sunrise, Switzerland), according to Bednar's method [12], changed by Chadderdon and Cappello [13], and was expressed in absolute value (mOD/min), which represent the mean decreasing of the optical density as a consequence of platelet aggregation induced by ADP. The platelet adhesion to fibrinogen was performed also with the microplate reader TECAN, according to Bellavite's method [14]. The results are expressed as percentage to the total number of platelets. The percentage of adherent cells was calculated on the basis of a standard curve.

### 2.6. Parameters of Lipid Profile

Serum total cholesterol, HDL-cholesterol, and triglycerides (TG) were measured by enzymatic colorimetric methods on a TECAN microplate reader by commercially available kits (Audit Diagnostics Ireland). Non-HDL cholesterol was calculated by subtracting HDL cholesterol from total cholesterol.

### 2.7. Morphological Study of Aorta

For light microscopy evaluation of aortic atherosclerotic lesions, we used hematoxylin-eosin (H-E) staining.

### 2.8. Statistical Analysis

Data were expressed as mean ± standard deviation (SD). Univariate statistical analysis was performed using the Student's *t*-test and Bonferroni's Multiple Comparison Test (Statistical Software Package SPSS, version 13, SPSS Incorporation, Chicago, IL, USA).

## 3. Results and Discussions

The literature's data supports the participation of the vascular wall in the atherogenesis process, involving an inflammatory process, endothelial dysfunction, and platelets hyperreactivity. Platelets receptors activation, platelet membrane fluidity alteration, and the membrane lipids composition change contribute to platelets activation. It has been shown that platelet activation is characterized by increased platelet adhesion and increased platelet aggregation, particularly to ADP, but also to thrombin or collagen [15].

In our research, the antiatherogenic mechanism of flaxseed enriched diet was investigated in ovariectomized female rats, a model of experimental atherosclerosis [1]. The absence of endogenous estrogens disturbs the lipid metabolism, decreases the antioxidant capacity, and alters the expression of adhesion molecules and platelet adhesion to endothelial cells [16, 17]. Moreover, the excess of saturated fatty acids determines hypercholesterolemia and could increase the atherogenic potential [17].

Table 2 shows the changes in platelet and endothelial markers by addition of lard or lard + flaxseed in Sham and Ovx groups. In our study, platelet aggregation significantly increased in lard-fed groups as compared to low-fat diet groups, while supplementing the diet with flaxseeds significantly decreased platelet aggregation only in Sham group. Lard diet resulted in significantly increased platelet adhesion in Ovx group and the addition of flaxseeds significantly decreased it. Serum sVCAM-1 increased in lard groups as compared to low-fat diet groups and the addition of flaxseeds significantly decreased this endothelial marker in Sham and Ovx groups. Serum vWF increased in Ovx groups as compared to Sham groups. The ovariectomized rats fed with lard + flaxseeds had significantly lower serum concentrations of vWF as compared to Ovx + lard group.

Table 3 shows the changes in serum lipid parameters by addition of lard or lard + flaxseed in Sham and Ovx groups. Ovariectomy significantly increased serum total cholesterol, non-HDL cholesterol, and TG. High-fat diet resulted in increased serum total cholesterol, non-HDL cholesterol, and TG as compared to low-fat diet in Sham groups. The flaxseed addition to the high-fat diet led to significant reduction of TG in Sham and Ovx groups. The supplementation of diet with flaxseed significantly decreased non-HDL cholesterol in Sham animals and nonsignificantly decreased total cholesterol and non-HDL cholesterol in ovariectomized female rats.

**Figure 1:**
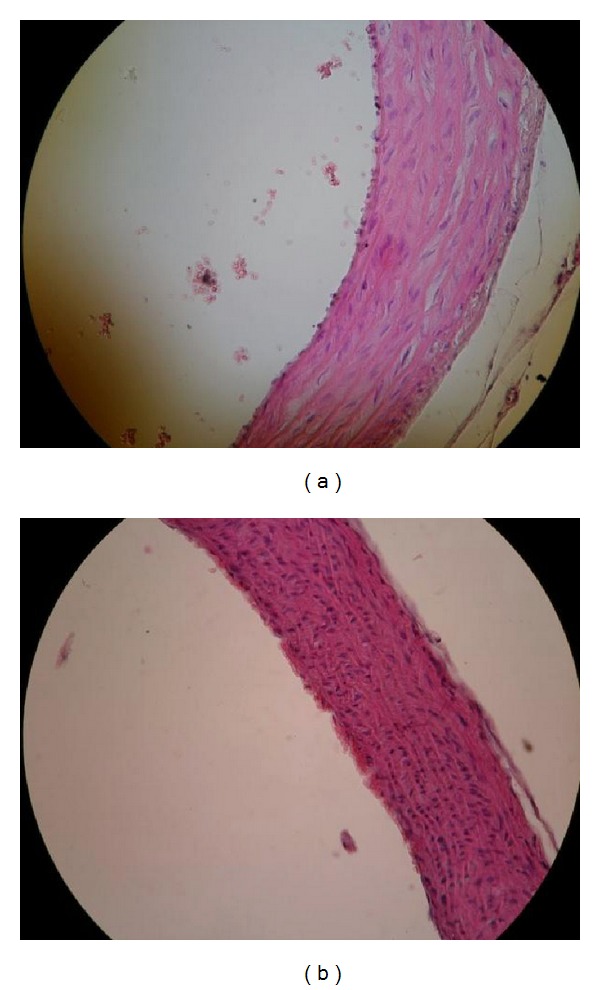
Aorta—Ovx + lard group (HE staining ×20). (a) Endothelitis. Adherent platelets. Leukocyte margination. (b) Isolated macrophages loaded with lipids in the internal third of the intima.

**Figure 2:**
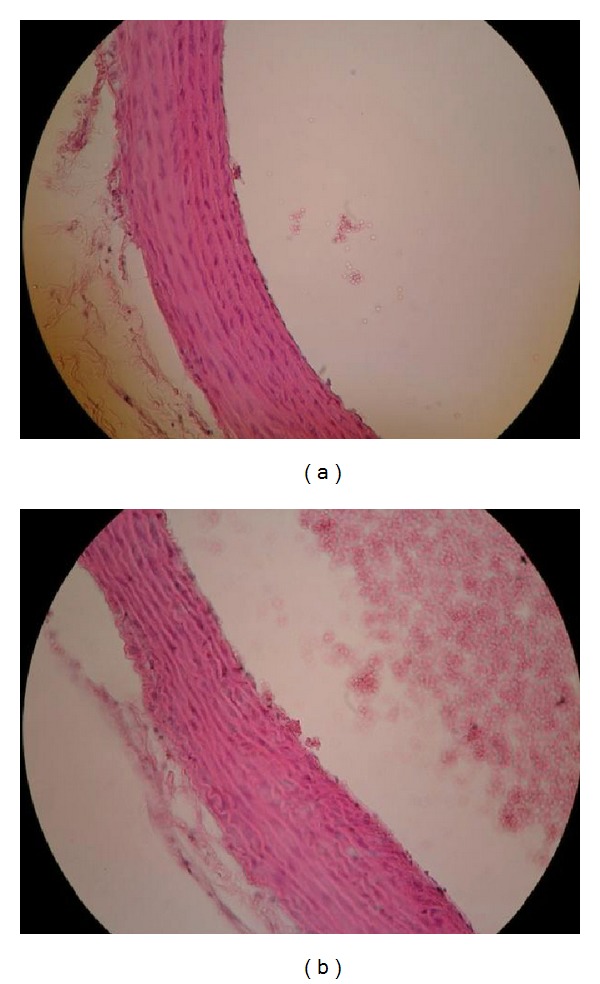
Aorta—Ovx + lard + flaxseed group (HE staining ×20). (a) Isolated adherent platelets. (b) Rare adherent platelets.

**Figure 3:**
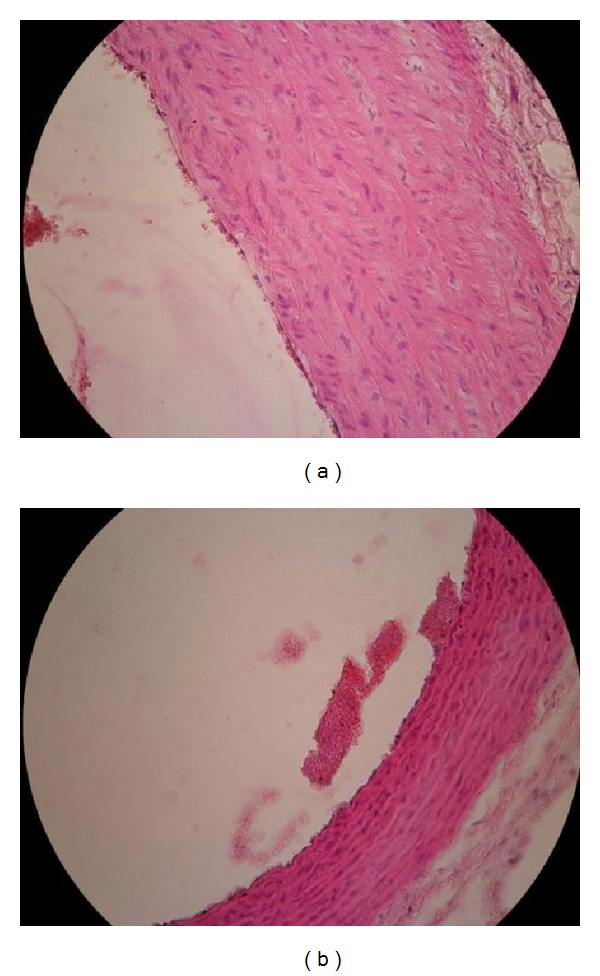
Aorta—Sham + lard + flaxseed group (HE staining ×20). (a) Rare adherent platelets. (b) No platelets adhered to the endothelium.

Examination of the aorta under a light microscope in lard-fed Ovx animals revealed signs of incipient atherosclerosis (endothelitis, leukocyte, and platelet adhesiveness and leukocyte margination, macrophage loaded with lipids in intima) (Figure 1). The histological evaluation of the aorta in ovariectomized group fed with lard + flaxseed diet showed a lower extent of platelet and leukocyte adherence to endothelium (Figure 2) similar to Sham group (Figure 3).

In the present study, ovariectomy and lard-based diet increased serum concentrations of total and non-HDL cholesterol, platelet aggregation and adhesion, and endothelial dysfunction markers (sVCAM-1 and vFW) and led to incipient atherosclerotic lesions in female rats fed on high-fat diet. The addition of ground flaxseed (15 g *Linum usitatissimum*/100 g food) to lard-based diet significantly reduced platelet adhesion and serum concentrations of endothelial integrity markers (vFW) and prevented the progression of atherosclerotic lesions in estrogen deficiency states. Our results clearly demonstrated that the flaxseed diet may protect against atherosclerotic lesions by decreasing platelet reactivity, without lowering effect on serum cholesterol.

The literature data on the hypocholesterolemic effect of flaxseeds are controversial. The experimental model of postmenopausal hypercholesterolemia and atherosclerosis, developed by Lucas et al. [1] on ovariectomized female hamsters, demonstrated that flaxseed was beneficial in reducing hypercholesterolemia and the progression of atherosclerotic lesions in ovarian hormone deficiency. Other studies [18], made on rabbits, showed that the formation of atherosclerotic lesions was decreased without a noticeable cholesterol lowering effect. Differences in response to supplementation with flaxseeds can be explained partly by species, age, and hormonal status of animals. The hypocholesterolemic effect of flaxseed could be attributed to flaxseed gum [19], to ALA [20], or to the lignan precursor present in flaxseed, (SDG) [21]. The lignans and the soluble fiber contained in flaxseeds may help lowering cholesterol [22]. These results suggested that the cardioprotective property of flaxseed could be due to its hypocholesterolemic effect but also to other potential mechanisms such as being antioxidative, anti-inflammatory, and/or antithrombotic.

Serum HDL-cholesterol changes in flaxseed-fed sham-operated and ovariectomized animals revealed that ALA and lignans supplements had beneficial effects by slight and nonsignificant increase in HDL-cholesterol as compared to lard-fed animals. Although the differences are not significant, it confirms the beneficial effects of flaxseeds, even when two risk factors are associated. Lignans from flaxseeds may play an important role in lipid metabolism modulation; synthetic lignans significantly reduce serum cholesterol and LDL cholesterol, while increasing HDL cholesterol; it is considered that lignans modulate 7*α*-hydroxylase and acyl-CoA cholesterol transferase activity, two key enzymes involved in cholesterol metabolism [23].

The results of our study suggested that in a condition associated with two cardiovascular risk factors, estrogen deficiency and increased saturated fatty acids intake, the endothelial markers and platelet functions are significantly changed and the diet supplementation with flaxseed had a beneficial effect.

The lack of estrogen atheroprotection in our animal model of atherosclerosis could be connected to the state of the NO endothelial production [1, 24]. The NO production would be involved in the mechanisms by which estrogens inhibit atherosclerosis and estrogen deficiency that are responsible for atherogenic changes in lipids, endothelial cells, smooth muscle cells, and platelets [25]. Moreover, in estrogen deficiency, the endothelial synthesis of NO is low, while superoxide anion concentrations are increased, destroying NO [15]. Since recent studies revealed that fish oil increased NO production and endothelial NO synthase (NOS) expression [26], the diet enriched in flaxseed may protect against increased platelet reactivity and endothelial dysfunction induced by estrogen deficiency [3].

High dose of flaxseed used in our study significantly reduced platelet adhesion in ovariectomized female rats. We have previously demonstrated that the addition of ground flaxseed (15 g *Linum usitatissimum*/100 g food) improved platelet functions and had antioxidative effect in ovariectomized hamsters [3]. The high flaxseed dose led to the enrichment of platelet membrane phospholipids with large amounts of eicosapentaenoic acid (EPA) and/or docosahexaenoic acid (DHA) [1, 25], resulting in decrease platelet reactivity or endothelial activation (by reducing sP-selectin concentrations) [27]. On the other hand, secoisolariciresinol diglucoside (SDG), isolated from flaxseed, has oxygen radical scavenging properties [28, 29] and inhibits lipid peroxidation [30], which could reduce oxidative changes of plasma lipoproteins and therefore decrease platelet adhesion to oxidized LDLs [31].

## 4. Conclusions

In our study, high dose of ground flaxseed incorporated to lard-based diet prevented the progression of atherosclerotic lesions in estrogen deficiency rats by decreasing platelet and endothelium reactivity without serum cholesterol lowering effect. Assessment of platelet adhesion, serum soluble adhesion molecule sVCAM, and endothelium integrity molecule vWF could be useful to detect the risk for atherosclerotic lesions in estrogen deficiency states and to estimate the effect of flaxseed supplementation.

Supplementing the diet with high doses of ground flaxseed may lower the atheroslerotic risk in postmenopausal women by increasing the vascular wall protection, reducing the thrombotic risk and improving the lipid metabolism.

## Figures and Tables

**Table 1:** Composition of experimental diets (%; w/w).

Experimental diets	8% fat	40% fat	40% fat
		Lard	Lard + flaxseed
Proteins	20.00	20.00	20.00
Corn starch	62.00	32.00	32.00
Cellulose powder	5.00	3.00	3.00
L-cysteine	0.25	0.25	0.25
Vitamin mix	1.00	1.00	1.00
Mineral mix	3.50	3.50	3.50
Choline	0.25	0.25	0.25
Fat	8.00	40.00	40.00
Sunflower oil	8.00	15.00	7.00
Lard	—	25.00	25.00
Flaxseeds*	—	—	8.00

*Flaxseeds (*Linum usitatissimum) *belonged to the Olin variety and were provided by the Department of Phytotechny, Faculty of Agronomy Iasi. The composition of flaxseeds was: 40.2% oil (55.6% linolenic acid) and 19.5% proteins.

**Table 2:** Mean ± SD values for platelet functions and endothelial markers in studied groups.

Measures	Sham	Sham + lard	Sham + lard + flaxseed	Ovx	Ovx + lard	Ovx + lard + flaxseed
Platelet functions						
Aggregation (mOD/min)	7.9 ± 1.2	21.0 ± 1.3^b^	14.6 ± 1.3^bc^	8.1 ± 1.2	18.8 ± 1.3^b^	15.6 ± 1.2^b^
Adhesion (%)	29 ± 6	34 ± 3	31 ± 4	31 ± 7	40 ± 9^b^	34 ± 4^c^

Endothelial markers						
sVCAM (ng/mL)	175 ± 64	286 ± 26^b^	294 ± 11^b^	252 ± 53^a^	539 ± 162^ab^	404 ± 10^ac^
vWF (%)	111 ± 6	118 ± 6	104 ± 10	178 ± 13^a^	193 ± 16^ab^	155 ± 10^ac^

Values are means ± SD, *n* = 7 in each group.

^a^ *P* < 0.05 as compared to corresponding Sham groups.

^b^ *P* < 0.05 as compared to groups fed with low-fat diet.

^c^ *P* < 0.05 between lard and lard + flaxseed fed groups.

**Table 3:** Mean ± SD values for lipid profile in studied groups.

Measures	Sham	Sham + lard	Sham + lard + flaxseed	Ovx	Ovx + lard	Ovx + lard + flaxseed
Serum						
Total cholesterol (mg/dL)	71 ± 4	92 ± 4^b^	72 ± 4^c^	104 ± 4^a^	104 ± 4	98 ± 4^a^
Triglycerides (mg/dL)	28 ± 9	94 ± 16^b^	40 ± 14^c^	80 ± 14^a^	81 ± 12	57 ± 15^abc^
HDL-cholesterol (mg/dL)	51 ± 10	52 ± 11	55 ± 11	61 ± 11	61 ± 12	65 ± 9
Non-HDL cholesterol (mg/dL)	20 ± 9	41 ± 12^b^	16 ± 3^c^	43 ± 16^a^	43 ± 18	33 ± 9^a^

Values are means ± SD, *n* = 7 in each group.

^a^ *P* < 0.05 as compared to corresponding Sham groups.

^b^ *P* < 0.05 as compared to groups fed with low-fat diet.

^c^ *P* < 0.05 between lard and lard + flaxseed fed groups.
